# Association study between immune-related miRNAs and mixed connective tissue disease

**DOI:** 10.1186/s13075-020-02403-9

**Published:** 2021-01-11

**Authors:** Barbara Stypińska, Aleksandra Lewandowska, Anna Felis-Giemza, Marzena Olesińska, Agnieszka Paradowska-Gorycka

**Affiliations:** 1 Department of Molecular Biology, National Institute of Geriatrics, Rheumatology, and Rehabilitation, Spartańska 1, 02-637 Warsaw, Poland; 2 Department of Connective Tissue Diseases, National Institute of Geriatrics, Rheumatology, and Rehabilitation, Spartanska 1, Warsaw, 02-637 Poland

**Keywords:** MCTD, MicroRNA, Single-nucleotide polymorphisms, SNPs, Pathogenesis, Epigenetics

## Abstract

**Background:**

Mixed connective tissue disease (MCTD) is a rare condition that is distinguished by the presence of specific U1-RNP antibodies. Information about its etiopathology and diagnostics is still unclear. miRNAs such as miR-146, miR-155, and miR-143 emerged as key regulators of the immune system, known to be involved in the development of autoimmune diseases and cancers. We performed an association study between immune-related miRNAs and MCTD severity and susceptibility.

**Methods:**

A total of 169 MCTD patients and 575 healthy subjects were recruited to the case–control study. The miRNA polymorphisms were genotyped using TaqMan SNP genotyping assay. TNF-α, IL-6, and IFN-γ levels in serum were determined using ELISA. qRT-PCR of TRAF6, IRAK1, and microRNAs was performed using Taqman miRNA assays and TaqMan Gene Expression Assays.

**Results:**

miR-146a rs2910164 G allele and GG genotype as well as miR-143 rs713147 A allele were more frequent in healthy subjects than in MCTD patients. miR-146a rs2910164 CC genotype and miR-143 T-rs353299*T-rs353291*T-rs713147*G-rs353298 and C-rs353299*C-rs353291*T-rs713147*A-rs353298 haplotypes were associated with MCTD susceptibility. miR-146a rs2910164 C/T was associated with scleroderma and lymphadenopathy. miR-143 rs353299 C/T was associated with swollen fingers or hands, the presence of enlarged lymph nodes, and pericarditis/pleuritis. miR-143 rs353298 A/G was associated with the occurrence of pericarditis/pleuritis and scleroderma. miR-143 rs353291 T/C showed association with pericarditis/pleuritis. The serum TNF-α, IFN-γ, and IL-6 levels were significantly higher in MCTD patients compared to healthy subjects. miR-143 SNPs were associated with higher proinflammatory cytokine concentration in serum only in healthy controls. IRAK1 and TRAF6 expression were higher in the MCTD patients compared to controls.

**Conclusions:**

The results of our case–control study indicate the possible significance of miR-146a and miR-143/145 in the susceptibility and clinical picture of MCTD.

**Supplementary Information:**

The online version contains supplementary material available at 10.1186/s13075-020-02403-9.

## Introduction

Autoimmunity, an abnormal response of the body to its own tissue antigens, is manifested in many ways that are categorized into various types of diseases depending on the tissue and antigen targeted. Mixed connective tissue disease (MCTD) is a rare disease entity that belongs to the so-called overlap syndromes. This means that it meets the diagnostic criteria for more than one autoimmune connective tissue disease (ACTD). MCTD combines the mild symptoms of systemic lupus erythematosus (SLE), systemic sclerosis (SSc), polymyositis/dermatomyositis (PM/DM), and rheumatoid arthritis (RA) with the increased titer of U1snRNP antibodies [1, 2]. Due to the controversy associated with this disease, there are no diagnostic criteria for MCTD, approved by ACR. However, there are several diagnostic criteria for this disease in the literature, published by Sharp, Alarcón-Segovia, Kasukawa, and Kahn [1]. The high titer of ANA and U1-RNP are the first clue in the diagnosis of MCTD (in the absence of other specific antibodies) [1, 2]. Scientific research on MCTD patients allows to answer the question about the differences and similarities in pathogenesis of MCTD compared to other ACTDs. Greater knowledge on this subject will allow to unequivocally state if MCTD is a separate disease entity. It will facilitate diagnosis and treatment of patients who at the moment cannot be assigned to any existing ACTD.

The exact cause for immune system dysregulation and chronic inflammation is ambiguous. A significant step in understanding the processes regulating the proper functioning of the immune system was the discovery of the microRNA (miRNA). miRNAs regulate the immune system and the immune response. Specific miRNAs modulate antigen presentation, clonal selection, Th skewing, Treg function, cytokine production, cytokine functions, recruitment of chemo-dependent inflammatory cells, antibody production, and non-immune cell mechanisms of tissue damage [3–5]. Diseases with the autoimmune background are characterized by altered miRNA expression patterns which are able to exacerbate disease severity [4, 6, 7]. Research can provide evidence that miRNA can be used as diagnostic and prognostic biomarkers and help in the classification of patients as well as in defining disease advancement and predict future outcome [6, 7]. Among many miRNAs, miR-146, miR-155, miR-143, and miR-145 emerged as key regulators of the immune system [8, 9]. MiR-146a is a negative feedback regulator of the innate and adaptive immune response. It regulates TLR signaling pathway and IFN type1 production. It also participates in the proper functioning of Treg cells and Th17-induced differentiation [8, 10–15]. MiR-155 facilitates cellular proinflammatory response. It is necessary for the proper immune cell activation and production of proinflammatory cytokines; its presence promotes cell survival, growth, migration, and anti-pathogenic response [8, 9, 15–19]. miR-143/miR-145 play an important role in intestinal physiology and cancer formation. It enhances proliferation, migration, and invasion of cancer cells [20–28]. Due to the great importance of these microRNAs in the functioning of the immune system and the relationship of these microRNAs with the occurrence of other ACTDs, assessing whether they are also associated with MCTD patients seems justified.

The aim of this work was an evaluation of the possible involvement of key to immune system miRNAs in MCTD. We performed genotype/haplotype association analysis to assess whether selected miRNA SNPs are associated with the incidence and clinical picture of MCTD. Moreover, we evaluated differences in serum miRNA expression in MCTD patients compared to healthy controls. We also analyzed if selected genotypes are connected with the serum concentration of proinflammatory cytokines in patients suffering from MCTD. To our knowledge, this is the first study concerning the epigenetic association with MCTD.

## Patient data materials and methods

### Study population

A total of 169 patients suffering from MCTD (84% of women and 16% of men with mean age 43.43 ± 13.77) diagnosed at the Clinic and Polyclinic of Connective Tissue Diseases of the National Institute of Geriatrics, Rheumatology and Rehabilitation in Warsaw and 575 healthy people (61.92% of women and 58.08% of men with mean age 37.29 ± 12.08) from Warsaw Center for Blood Donation were recruited to the case–control study (Caucasian origin). Patients met the diagnostic criteria of Kasukawa and/or Alarcon-Segovia and Villarreal. Healthy subjects did not have a history of autoimmune and/or inflammatory disease at the time of sampling. Autoantibodies to dsDNA, Scl-70, Sm, Ro, La, RibP, His, PCNA, CENPB, and Jo-1 were determined in the serum using DOT-blot tests (recomLine ANA/ENA, Mikrogen Diagnostik, Neuried, Germany). The identification of anti-U1-RNP was performed by electrochemiluminescence (ECLIA) using streptavidin-coated paramagnetic beads (UNICAP100, Phadia, Sweden). The presence of antinuclear antibodies (ANA) was determined by indirect immunofluorescence (IF) on Hep2 cell lines (Euroimmun Polska, Wroclaw, Poland), with a median titer of 1:5840 (range 1:80–1:40,960).

All participants signed informed written consent for participation in the study. The study was approved by the ethics committee in the National Institute of Geriatrics Rheumatology and Rehabilitation, Warsaw, Poland.

### Genotyping

Genomic DNA extraction was performed from peripheral blood using either the salting-out procedure or DNA isolation kit (Qiagen). Allelic discrimination of miR-155 rs1893650, rs2829806, miR-143 rs353299, rs353291, rs713147, rs353298, and miR-146a rs2910164 (Supplementary Table 1) was performed using TaqMan® probes (Applied Biosystems, Carlsbad, CA, USA) on the QuantStudio 5 Real-Time PCR System (ThermoFisher Scientific, USA). The reaction was performed in a total volume of 10 μl, and reaction conditions were as follows: denaturation at 95 °C for 10 min, followed by 40 cycles of denaturation at 92 °C for 15 s, and annealing and extension at 60 °C for 1 min. The first step of SNP selection for the analysis was based on the 1000 genome EUR, ExAC Europe, or HapMap CEU Data base. We choose only this SNP, which minor allele frequency was greater than 0.02. The second stage was based on the literature; we decided to choose SNP examined earlier in the context of connective tissue diseases.

### Assay for serum levels of TNF-α, IL-6, and IFN-γ

TNF-α, IL-6, and IFN-γ protein concentrations, in the serum of 100 randomly selected MCTD patients and 130 healthy subjects, were determined using ELISA DRG diagnostics immunosorbent assays (REF: EIA-4641, REF: EIA-4640, and REF: EIA-4434 respectively) (DRG International, Inc. USA), following the manufacturer’s instructions. Plates were read on a microplate reader (El × 800, BIO-TEK Winooski, United States Instruments).

### miRNA relative expression

The expression level of miRNAs in the serum was determined using qRT-PCR in 22 MCTD patients and 38 healthy controls. miRNA from 200-μl freshly isolated serum was extracted using AA Biotech MicroRNA Concentrator (A&A Biotechnology, Poland) according to the manufacturer’s protocol. Multiplexed reverse transcription and preamplification reactions were performed according to the Protocol for Creating Custom RT and Preamplification (Publication Part Number 4465407 Revision Date January 2013 (Rev. C), Applied Biosystems by Life Technologies); Taqman miRNA assays (miR-143 TM:002249, miR-145 TM:002278, miR-155 TM:002623, U6 – TM:001973) were used to determine the expression of selected miRNAs following the manufacturer’s instructions. Each target was measured in triplicate and normalized to the level of U6.

### IRAK1 and TRAF6 mRNA relative expression

Total RNA was isolated from whole blood using AA Biotech MicroRNA Concentrator (A&A Biotechnology, Poland). cDNA was obtained using the High Capacity cDNA Reverse Transcription Kit with RNase Inhibitor (Applied Biosystems, Foster City, CA). qPCR was performed in 45 MCTD patients and 49 healthy controls using TaqMan Gene Expression Assays (IRAK1 HS00155570_m1 and TRAF6 – Hs00377558_m1) (Applied Biosystems, Foster City, CA) according to manufacturer’s instructions. Each target was measured in triplicate and normalized to the level of GAPDH.

### Statistical analysis

The results were presented as a median and interquartile range (IQR) for non-normally distributed continuous variables or mean with one standard deviation for normally distributed continuous variables. The consistency of genotype distribution with Hardy–Weinberg equilibrium (HWE) was performed using the HWE exact test with “genetics” R package (Gregory Warnes, with contributions from Gregor Gorjanc, Friedrich Leisch and Michael Man (2019) genetics: Population Genetics. R package version 1.3.8.1.1. https://CRAN.R-project.org/package). Genotype and allele distribution between groups was evaluated using logistic regression (OR, 95% confidence intervals, *p* value). The analysis considered the effect of possible confounders like age and gender. The analysis was calculated under dominant, codominant, overdominant and recessive, and allelic models. The presence of LD for haplotypes and LD heatmap were determined using package “genetics” and “LDheatmap” R packages. Differences between tested SNPs, disease activity parameters, and cytokine serum concentrations were analyzed using the Kruskal–Wallis test, Mann–Whitney test, or analysis of variance for continuous variables and *χ*^2^ or Fisher exact test for categorical variables. A *p* value < 0.05 indicated a statistically significant result. Only for haplotypes Bonferroni correction was used to adjust the significance of *p* value for multiple testing. The relative expression level of miRNAs was computed using the 2−ΔΔCt method (Livak) with normalization to the endogenous small RNA control, U6. Effect size for the Kruskal–Wallis test was presented by *ε* square [29]. The statistical analysis and figure preparation were carried out using the R program (HTTP: //www.R-project.org.) supplemented with the following packages: Gregory Warnes, with contributions from Gregor Gorjanc, Friedrich Leisch, and Michael Man (2019). genetics: Population Genetics. R package version 1.3.8.1.1. https://CRAN.R-project.org/package=genetics; Juan R González, Lluís Armengol, Elisabet Guinó, Xavier Solé, and Víctor Moreno (2014). SNPassoc: SNPs-based whole genome association studies. R package version 1.9-2. https://CRAN.R-project.org/package=SNPassoc; H. Wickham. ggplot2: Elegant Graphics for Data Analysis. Springer-Verlag New York, 2016. Ogle, D.H., P. Wheeler, and A. Dinno. 2018. FSA: Fisheries Stock Analysis. R package version 0.8.22, https://github.com/droglenc/FSA; Sinnwell JP and Schaid DJ (2018). haplo.stats: Statistical Analysis of Haplotypes with Traits and Covariates when Linkage Phase is Ambiguous. R package version 1.7.9. https://CRAN.R-project.org/package=haplo.stats

## Results

### MCTD patients’ clinical characteristics

The demographic and clinical characteristics of our MCTD patients are presented in Table 1. MCTD patients who met the classification criteria for two different CTDs at the blood sampling were excluded from the study. The most common symptoms of the MCTD activity were as follows: swelling fingers or hands (92% of MCTD patients), decreased number of leukocytes and/or platelets, increased ESR and/or CRP levels, hypergammaglobulinemia, and skin rashes. Damage most commonly affected the skin, musculoskeletal and cardiovascular systems, and lungs. The clinical picture of MCTD is very variable. Almost all MCTD patients presented with Raynaud’s phenomenon (97% of MCTD patients) right at the onset of the disease. Pulmonary arterial hypertension (PAH), routinely screened in MCTD patients, was detected in 31% of our patients. Antinuclear antibodies (ANA) in titer > 1:320 were detected in 99% of patients with MCTD. All our MCTD patients had anti-U1-RNP antibodies, where anti-70K was detected in 75% of patients, anti-A in 83% of patients, and anti-C in 79% of patients. Patients’ therapeutic profile presented as follows: immunosuppressive drugs (azathioprine 6%), methotrexate (23%), corticosteroids (prednisone 77%), and antimalarics (chloroquine 59%).

**Table 1:** Clinical characteristics of patients with MCTD

MCTD				
Clinical parameters			N*	Mean values ± SD or n** (%)
**Demographic characteristics**	Age		*100*	43.43 ± 13.77471
	Gender (*n* = female)		*100*	84 (84%)
Disease activity	Disease duration [months]		*100*	148.2 ± 272.6612
	Damage index		*100*	9.79 ± 13.547
	The course of disease	Single phase	*100*	12 (12%)
		Multiphase		28 (28%)
		Progressive		60 (60%)
Autoantibody profile	Anti-U1 RNP		*100*	99 (99%)
	Anti-U1 RNP	70	*100*	75 (75%)
		A	*100*	83 (83%)
		C	*100*	79 (79%)
	Anti-CCP		*71*	3 (4.22%)
	Anti-ANA		*99*	98 (98.98%)
	Anti-SmB		*100*	31 (31%)
	Anti-SmD		*100*	6 (6%)
	Anti-Ro 60		*100*	10 (10%)
	Anti-Ro 52		*100*	24 (24%)
	Anti-La		*100*	2 (2%)
	Anti-Rib P		*100*	3 (3%)
	Anti-PCNA		*100*	1 (1%)
	Anti-CENP B		*100*	0
	Anti-Scl-70		*100*	2 (2%)
	Anti-Jo-1		*100*	1 (1%)
	Anti-His		*100*	15 (15%)
	Anti-DNA		*100*	13 (13%)
	RF		*98*	36 (36.73%)
Clinical manifestation	SLE-like skin lesions		*100*	71 (71%)
	Ocular lesions		*99*	60 (60.60%)
	Cardiomyopathy		*100*	4 (4%)
	Dysphagia		*100*	22 (22%)
	Deforming or erosive arthritis		*100*	12 (13%)
	Poikiloderma		*100*	31 (31%)
	Raynaud syndrome		*100*	97 (97%)
	Sjögren’s syndrome		*69*	22 (31.88%)
	Swollen fingers or hands		*100*	92 (92%)
	Polyarthritis		*100*	94 (94%)
	Lymphadenopathy		*100*	26 (26%)
	Facial erythema		*100*	43 (43%)
	Pericarditis/pleuritis		*100*	16 (16%)
	Leukopenia/thrombocytopenia		*100*	65 (65%)
	Scleroderma		*100*	34 (34%)
	Pulmonary fibrosis		*100*	31 (31%)
	Esophageal disorders		*100*	23 (23%)
	Myopathy		*100*	66 (66%)

*RF* rheumatoid factor, *CCP* anti-CCP antibodies, *U1 RNP* anti-U1RNP antibodies, *ANA* antinuclear antibodies, *70* anti-U1 70 kDa antibodies, *A* anti-U1 A antibodies, *C* anti-U1 C antibodies, *SmB* anti-Sjögren’s syndrome type B antibodies, *SmD* anti-SmD antibody, *Ro 60* anti-Ro-60 antibody, *Ro 52* anti-Ro52 antibody, *La* antibody to the La antigen, *Rib P* anti-ribosomal P protein antibody, *PCNA* anti-PCNA antibody, *CENP B* anti-CENP-B antibody, *Scl-70* anti-Scl-70 antibodies, *Jo-1* anti-Jo-1 antibody, *His* anti-histone antibody, *DNA* anti-dsDNA antibody

*N, number of patients with clinical information

**n, number of patients with positive clinical manifestation

### miRNA genotype distribution between MCTD patients and healthy subjects

We performed genotype distribution analysis, for two miR-155 SNPs (rs1893650 and rs2829806), four miR-143 SNPs (rs353291, rs353298, rs353299, rs713147), and one miR-146a (rs2910164), between MCTD and healthy controls. All examined SNPs were consistent with the Hardy–Weinberg equilibrium (HWE) (*p* > 0.05, Supplementary Table 1). The minor allele frequency (MAF) of all examined SNPs in our subjects, MCTD patients, and healthy controls were similar to those in the Utah Residents (CEPH) with Northern and Western Ancestry and in the European ancestry.

miRNA genetic variant distribution was calculated under dominant, codominant, overdominant, recessive, and allelic models (Table 2). We observed only a tendency where miR-146a rs2910164 G allele and GG genotype were more frequent in healthy subjects than in MCTD patients (81% vs 75% and 65% vs 56%, respectively). Nevertheless, differences observed in miR-146a rs2910164 codominant (GG vs GC vs CC) (*p* = 0.07) and overdominant (GC vs CC + GG) (*p* = 0.09) models were not statistically significant. We also observed that miR-143 rs713147 A allele has shown a tendency to more frequent occurrence in healthy subjects than in MCTD patients, thus conferring disease protection nature to this allele. Distribution of other examined SNPs did not show statistically significant differences between studied groups.

**Table 2:** Distribution of genotypes and allele frequencies of miRNA SNPs among patients with MCTD and healthy subjects (*p* = MCTD vs controls)

SNP	Genotype	MCTD ***n*** (%)	Controls ***n*** (%)	Adjusted OR (95% CI)	***p*** value
**miR-155 rs1893650 C/T**					
Codominant	CC	79 (56.43)	333 (62.95)	–	–
	CT	57 (40.71)	173 (32.70)	1.42 (0.89–2.27)	0.1
	TT	4 (2.86)	23 (4.35)	0.82 (0.19–2.62)	0.8
Dominant	CC	79 (56.43)	333 (62.95)	–	–
	TT+CT	61 (43.57)	196 (37.05)	1.35 (0.86–2.13)	0.2
Recessive	CC+CT	136 (97.14)	506 (95.65)	–	–
	TT	4 (2.86)	23 (4.35)	0.73 (0.16–2.25)	0.6
Overdominant	TT+CC	83 (59.29)	356 (67.30)	–	–
	CT	57 (40.71)	173 (32.70)	1.44 (0.90–2.27)	0.1
Alleles	C	215 (76.79)	839 (79.3)	–	–
	T	65 (23.21)	219 (20.7)	1.16 (1.59–0.85)	0.36
**miR-155 rs2829806 G/T**					
Codominant	GG	71 (59.66)	285 (61.69)		–
	GT	44 (36.9)	153 (33.12)	1.29 (0.77–2.13)	0.3
	TT	4 (3.3)	24 (5.19)	0.63 (0.13–2.12)	0.5
Dominant	GG	71 (59.6)	285 (61.69)	–	–
	TT+GT	48 (40.4)	177 (38.31)	1.2 (0.73–1.95)	0.4
Recessive	CC+GT	115 (96.6)	438 (94.8)	–	–
	TT	4 (3.36)	24 (5.19)	0.58 (0.12–1.89)	0.4
Overdominant	TT+GG	75 (63.03)	309 (66.88)	–	–
	GT	44 (36.97)	153 (33.12)	1.33 (0.80–2.17)	0.2
Alleles	T	52 (21.85)	201 (21.75)	–	–
	G	186 (78.15)	723 (78.25)	1 (1.42–0.71)	0.9
**miR-146a rs29101164 G/C**					
Codominant	GG	69 (55.65)	316 (64.89)	–	–
	GC	51 (41.12)	153 (31.42)	1.47 (0.74–8.68)	0.1
	CC	4 (3.23)	18 (3.7)	2.80 (0.89–2.41)	**0.09**
Dominant	GG	69 (55.65)	316 (64.89)	–	–
	CC+GC	55 (44.35)	171 (35.11)	1.55 (0.96–2.51)	**0.07**
Recessive	GG+GC	120 (96.77)	469 (96.30)	–	–
	CC	4 (3.23)	18 (3.7)	2.43 (0.65–7.36)	0.1
Overdominant	CC+GG	73 (58.87)	334 (68.58)	–	–
	GC	51 (41.12)	153 (31.42)	1.40 (0.85–2.26)	0.2
Alleles	C	59 (24.58)	189 (19.40)	–	–
	G	181 (75.42)	785 (80.60)	1.35 (1.89–0.97)	**0.08**
**miR-143 rs353291 T/C**					
Codominant	TT	47 (39.50)	140 (36.46)	–	–
	TC	51 (42.86)	184 (47.92)	0.95 (0.55–1.66)	0.8
	CC	21 (17.65)	60 (15.63)	1.17 (0.57–2.35)	0.6
Dominant	TT	47 (39.50)	140 (36.46)	–	–
	CC +CT	72 (60.5)	244 (63.54)	1.01 (0.60–1.70)	0.9
Recessive	TT+CT	98 (82.35)	324 (84.37)	–	–
	CC	21 (17.65)	60 (15.63)	1.2 (0.62–2.24)	0.5
Overdominant	TT+CC	68 (57.14)	200 (52.08)	–	–
	TC	51 (42.86)	184 (47.92)	0.91 (0.55–1.48)	0.7
Alleles	T	145 (60.92)	464 (60.42)	–	–
	C	93 (39.08)	304 (39.58)	0.97 (1.32–0.73)	0.9
**miR-143 rs713147 T/A**					
Codominant	TT	63 (54.31)	148 (44.31)	–	–
	TA	44 (37.93)	148 (44.31)	0.72 (0.41–1.23)	0.2
	AA	9 (7.76)	38 (11.38)	0.52 (0.19–1.27)	0.1
Dominant	TT	63 (54.31)	148 (44.31)	–	–
	AA +TA	53 (45.69)	186 (55.69)	0.68 (0.40–1.12)	0.1
Recessive	TT+TA	107 (92.24)	276 (88.62)	–	–
	AA	9 (7.76)	38 (11.38)	0.61 (0.23–1.42)	0.2
Overdominant	TT+AA	72 (62.07)	186 (55.69)	–	–
	TA	44 (37.93)	148 (44.31)	0.80 (0.47–1.34)	0.4
Alleles	T	170 (73.28)	444 (66.47)	–	–
	A	62 (26.72)	224 (33.53)	0.72 (1–0.52)	**0.06**
**miR-143 rs353298 A/G**					
Codominant	AA	47 (39.17)	179 (37.61)	–	–
	AG	53 (44.17)	233 (48.95)	0.95 (0.56–1.61)	0.8
	GG	20 (16.17)	64 (13.45)	1.32 (0.64–2.62)	0.4
Dominant	AA	47 (39.17)	179 (37.61)	–	–
	GG +AG	73 (60.83)	297 (62.39)	1.03 (0.63–1.70)	0.8
Recessive	AA+AG	100 (83.33)	412 (86.55)	–	–
	GG	20 (16.17)	64 (13.45)	1.36 (0.70–2.51)	0.3
Overdominant	AA+GG	67 (55.83)	243 (51.05)	–	–
	AG	53 (44.17)	233 (48.95)	0.87 (0.53–1.40)	0.5
Alleles	A	147 (61.25)	591 (62.08)	–	–
	G	93 (38.75)	361 (37.92)	1.04 (1.39–0.77)	0.81
**miR-143 rs353299 C/T**					
Codominant	CC	47 (38.52)	130 (38.92)	–	–
	TC	56 (45.90)	162 (48.50)	1.13 (0.64–1.98)	0.6
	TT	19 (15.57)	42 (12.57)	1.35 (0.61–2.93)	0.4
Dominant	CC	47 (38.52)	130 (38.92)	–	–
	TT+CT	75 (38.52)	204 (38.92)	1.18 (0.69–2.01)	0.5
Recessive	CC+CT	103 (84.43)	292 (87.43)	–	–
	TT	19 (15.57)	42 (12.57)	1.27 (0.60–2.54)	0.5
Overdominant	TT+CC	66 (54.1)	172 (51.5)	–	–
	TC	56 (45.90)	162 (48.50)	1.04 (0.62–1.72)	0.8
Alleles	C	150 (61.48)	422 (63.17)	–	–
	T	94 (38.52)	246 (36.83)	1.08 (1.45–0.79)	0.64

*MCTD* mixed connective tissue diseases, *n* number of observations, *OR* odds ratio, *CI* confidence interval; *p* value obtained from linear regression, adjusted for gender and age; *p* value ≤ 0.05 was considered significant

### Haplotype association with the risk of MCTD

In the next step, we investigated the genetic association between miRNA haplotypes and MCTD. To create the haplotypes, miR-143 SNPs were analyzed in the following sequence: rs353299, rs353291, rs713147, and rs353298. Haplotypes with frequency < 0.03, in both examined groups, were ignored. We observed that two miR-143 haplotypes TTTG and CCTA occur much more frequently in MCTD patients than in healthy people (31% vs 14%, *p* < 0.001 and 23% vs 11%, *p* = 0.002 respectively) (Table 3). In contrast, the miR-143 haplotypes CTTG, TTAA, TCTA, and TTTA occur much more frequently in healthy subjects than in MCTD patients (7% vs 1%, 4% vs 0%, 6% vs 1%, and 5% vs 1%, respectively). Haplotype analysis of miRNA-155 did not show differences between study groups (data not shown). The strongest LD was observed between miR143 rs353298 and rs353299 *D*’ = 0.7, *r*^2^ = 0.5; LD between other miR143 variants was low (Supplementary Figure 1).

**Table 3:** miR-143 haplotype distribution

rs353299	rs353291	rs713147	rs353298	***p*** value	Hap-Freq Healthy control	Hap-Freq MCTD
**T**	T	T	G	**< 0.0001**	0.14	0.31
**C**	C	T	A	**0.002**	0.11	0.23
**C**	T	T	G	0.004	0.07	0.01
**T**	T	A	A	0.008	0.04	NA
**T**	C	T	A	0.009	0.06	0.01
**T**	T	T	A	0.02	0.05	0.01
**C**	C	T	G	0.16	0.04	0.02

*MCTD* mixed connective tissue diseases, *Hap-Freq* haplotype frequency; *2n*_*MCTD*_*-210* 2n_Control_-470 Bonferroni correction was used to adjust the significance of *p* value. *p* value < 0.003 was considered as significant

### Association of the miR-155, miR-143, and miR-146a genetic variants with the clinical features of MCTD patients

We evaluated an association between the miR-155, miR-146a, and miR-143 genotypes and haplotypes and the occurrence of individual clinical symptoms of MCTD. Analysis was performed on 100 MCTD patients with detailed clinical description. Our analysis indicated that miR-146a and miR-143 gene polymorphisms are associated with some clinical parameters indicating a more severe course of the disease.

miR-146a rs2910164 CC genotype and C allele were associated with scleroderma (Table 4). Recessive (*p* = 0.01, OR = 18.74), codominant (*p* = 0.04, OR = 22.94), and allelic (*p* = 0.02, OR = 2.17) models showed that among miR-146a rs2910164 CC carriers, scleroderma was much more common. In contrary, miR-146a rs2910164 CC and GC genotypes and C allele occurred less frequent among MCTD patients with lymphadenopathy. It has to be noted that among the patients for whom we had information on, only 4 people have the CC genotype.

**Table 4:** MCTD clinical parameters significantly associated with miR-146a rs2910164 G/C

***Scleroderma***								
**miR-146a rs2910164 G/C**	***n*** **(%)**			**Model**	*p*	OR (95% CI)	95% CI	
**Genotype**	**GG**	**GC**	**CC**	**CC + GC vs GG**	0.14	2.06 (0.88–4.8)		
Scleroderma	15 (27.78)	15 (38)	4 (100)	**GG + GC vs CC**	**0.01**	18.74	0.9773	359.2678
Without scleroderma	39 (72.22)	24 (62)	0 (0)	**CC + GG vs GC**	0.72	1.28	0.55	2.99
**Allele**	**G**	**C**		**CC vs GG**	**0.04**	22.94	1.1646	451.6701
Scleroderma	45 (31)	23 (49)		**GC vs GG**	0.27	1.63	0.68	3.91
Without scleroderma	102 (69)	24 (51)		**C vs G**	**0.02**	2.17	1.11	4.25
***Lymphadenopathy***								
**miR-146a rs2910164 G/C**	***n*** **(%)**			Model	*p*	OR	95% CI	
**Genotype**	**GG**	**GC**	**CC**	**CC + GC vs GG**	**0.005**	0.21	0.07	0.61
Lymphadenopathy	21 (38.89)	5 (13)	0 (0)	**GG + GC vs CC**	0.57	0.283	0.0147	5.4407
Without lymphadenopathy	33 (61.11)	34 (87)	4 (100)	**CC + GG vs GC**	**0.01**	0.26	0.09	0.76
**Allele**	**G**	**C**		**CC vs GG**	0.24	0.1731	0.0089	3.3799
Lymphadenopathy	47 (32)	5 (11)		**GC vs GG**	**0.008**	0.23	0.08	0.68
Without lymphadenopathy	100 (68)	42 (89)		**C vs G**	**0.006**	0.25	0.09	0.68

*n* number of patients with clinical manifestation; *p*< 0.05 is considered significant, indicated in bold; *p* value obtained from *χ*^2^ or Fisher exact test

Association analysis of miR-143 rs353299 C/T polymorphism showed that among MCTD patients with miR-143 rs353299 CT genotype, swollen fingers or hands, the presence of enlarged lymph nodes, and pericarditis/pleuritic were observed more often compare to MCTD patients with other miR-143 rs353299 genotypes (Table 5). On the other hand, the miR-143 rs353299 TT genotype appears to be protective, although differences were not significant. Within MCTD patients carrying miR-143 rs353299 TT genotype, swollen fingers or hands (*p* = 0.07 recessive model) and lymphadenopathy (*p* = 0.06 recessive model) were observed less than in MCTD patients with other miR-143 rs353299 genotypes.

**Table 5:** Association between miR-143 rs353299 C/T polymorphism and MCTD clinical parameters

***Swollen fingers or hands***								
**miR-143 rs353299 C/T**	***n*** **(%)**			**Model**	***p***	**OR**	**95% CI**	
**Genotype**	**CC**	**CT**	**TT**	**TT + CT vs CC**	0.25	1.87	0.36	9.83
Swollen fingers or hands	31 (91.18)	46 (100)	12 (80)	**CC + CT vs TT**	**0.07**	0.16	0.03	0.86
Without swollen fingers or hands	3 (8.82)	0 (0)	3 (20)	**TT + CC vs CT**	**0.07**	13.8966	0.7600	254.09
**Allele**	**C**	**T**		**TT vs CC**	0.28	0.39	0.07	2.19
Swollen fingers or hands	108 (95)	70 (92)		**CT vs CC**	0.12	10.33	0.5157	207.0449
Without swollen fingers or hands	6 (5)	6 (8)		**C vs T**	0.4679	0.65	0.2	2.09
***Lymphadenopathy***								
**miR-143 rs353299 C/T**	***n*** **(%)**			**Model**	***p***	**OR**	**95% CI**	
**Genotype**	**CC**	**CT**	**TT**	**TT + CT vs CC**	0.35	1.81	0.67	4.87
Lymphadenopathy	7 (20)	18 (39)	1 (7)	**CC + CT vs TT**	**0.06**	0.16	0.02	1.28
Without lymphadenopathy	28 (80)	28 (61)	14 (93)	**TT + CC vs CT**	**0.02**	3.38	1.29	8.82
**Allele**	**C**	**T**		**TT vs CC**	0.26	0.29	0.03	2.56
Lymphadenopathy	32 (28)	20 (26)		**CT vs CC**	**0.07**	2.57	0.93	7.12
Without lymphadenopathy	84 (72)	56 (74)		**C vs T**	0.84	0.94	0.49	1.8
***Pericarditis/pleuritis***								
**miR-143 rs353299 C/T**	***n*** **(%)**			**Model**	***p***	**OR**	**95% CI**	
**Genotype**	**CC**	**CT**	**TT**	**TT + CT vs CC**	0.85	20.14	6.58	61.68
Pericarditis/pleuritis	5 (14.29)	41 (89)	6 (40)	**CC + CT vs TT**	**0.02**	0.51	0.17	1.56
Without pericarditis/pleuritis	30 (85.71)	5 (11)	9 (60)	**TT + CC vs CT**	0.18	29.07	9.26	91.31
**Allele**	**C**	**T**		**TT vs CC**	**0.05**	4	0.99	16.24
Pericarditis/pleuritis	51 (44)	53 (70)		**CT vs CC**	**< 0.0001**	49.2	13.06	185.29
Without pericarditis/pleuritis	65 (56)	23 (30)		**C vs T**	**0.0006**	2.94	1.59	5.41

*n* number of patients with clinical information; *p* value < 0.05 is considered significant, indicated in bold; *p* value obtained from *χ*^2^ or Fisher exact test

Our analysis showed that miR-143 rs353298 A/G genetic variant was associated with the occurrence of pericarditis/pleuritis and scleroderma (Table 6). The miR-143 rs353298 GG genotype occurrence was associated with pericarditis/pleuritis symptom. The results indicated that MCTD patients who were GG homozygotes much more often suffered from pericarditis/pleuritis than in the MCTD patient with other genotypes (35% vs 12%, *p* = 0.04 recessive model, *p* = 0.04 codominant model). On the other hand, MCTD patients with miR-143 rs353298 GG genotype were less likely to experience scleroderma than MCTD patients with other genotypes (recessive model *p* = 0.05).

**Table 6:** Association between miR-143 rs353298 A/G polymorphism and MCTD clinical parameters

***Pericarditis/pleuritis***								
**miR-143 rs353298 A/G**	***n*** **(%)**			**Model**	***p***	**OR**	**95% CI**	
**Genotype**	**AA**	**AG**	**GG**	**GG + AG vs AA**	0.49	1.83	0.54	6.26
Pericarditis/pleuritis	4 (11.11)	5 (12)	6 (35)	**AA + AG vs GG**	**0.04**	4.18	1.24	14.07
Without pericarditis/pleuritis	32 (88.89)	37 (88)	11 (65)	**GG + AA vs AG**	0.52	0.58	0.18	1.85
**Allele**	**A**	**G**		**GG vs AA**	**0.04**	4.36	1.04	18.39
Pericarditis/pleuritis	13 (11)	17 (22)		**AG vs AA**	0.9129	1.08	0.27	4.37
Without pericarditis/pleuritis	101 (89)	59 (78)		**G vs A**	**0.05**	2.24	1.02	4.93
***Scleroderma***								
**miR-143 rs353298 A/G**	***n*** **(%)**			**Model**	***p***	**OR**	**95% CI**	
**Genotype**	**AA**	**AG**	**GG**	**GG + AG vs AA**	0.87	0.84	0.35	2.01
Scleroderma	13 (36.11)	17 (40)	2 (12)	**AA + AG vs GG**	**0.05**	0.21	0.05	1
Without scleroderma	23 (63.89)	25 (60)	15 (88)	**GG + AA vs AG**	0.30	1.72	0.73	4.06
**Allele**	**A**	**G**		**GG vs AA**	0.0814	0.24	0.05	1.2
Scleroderma	43 (38)	21 (28)		**AG vs AA**	0.6929	1.2	0.48	3.01
Without scleroderma	71 (62)	55 (72)		**A vs G**	0.1509	0.63	0.34	1.18

*n* number of patients with clinical information; *p* value < 0.05 is considered significant, indicated in bold; *p* value obtained from *χ*^2^ or Fisher exact test

The miR-143 rs353291 T/C gene polymorphism showed association only with pericarditis/pleuritis (Table 7). We observed that this symptom occurs less frequent in MCTD patients with miR-143 rs353291 TC genotype (*p* = 0.03 overdominant model).

**Table 7:** MCTD clinical parameters significantly associated with miR-143 rs353291 T/C

***Pericarditis/pleuritis***								
miR-143 rs353291 T/C	***n*** (%)			Model	***p***	OR	95% CI	
**Genotype**	**TT**	**TC**	**CC**	**CC + TC vs TT**	0.33	0.54	0.18	1.59
Pericarditis/pleuritis	8 (22.22)	3 (7)	5 (28)	**TT + TC vs CC**	0.18	2.34	0.7	7.88
Without pericarditis/pleuritis	28 (77.78)	39 (93)	13 (72)	**CC + TT vs TC**	**0.03**	0.24	0.06	0.92
**Allele**	**T**	**C**		**CC vs TT**	0.6532	1.35	0.37	4.92
Pericarditis/pleuritis	19 (17)	13 (17)		**TC vs TT**	0.0687	0.27	0.07	1.11
Without pericarditis/pleuritis	95 (83)	65 (83)		**T vs C**	1	1	0.46	2.17

*n* number of patients with clinical information; *p* value < 0.05 is considered significant, indicated in bold; *p* value obtained from *χ*^2^ or Fisher exact test

### Impact of miRNA polymorphisms on inflammatory cytokine levels in serum

miRNAs, which are critical in the development of the immune response, are involved in controlling genes associated with cytokine secretion and regulation of the cytokine serum levels. Therefore, we decided to check if examined miRNA polymorphisms may have an impact on the levels of the inflammatory cytokine levels in serum in our study groups. IL-6, TNF-α, and IFN-γ levels in serum were assessed in both MCTD patients and healthy subjects.

First, we compared TNF-α, IFN-γ, and IL-6 levels in serum between MCTD patients and healthy subjects (data not shown). We found that serum TNF-α, IFN-γ, and IL-6 levels were significantly higher in MCTD patients compared to healthy subjects (*p* < 0.001 in each case).

**Fig. 1:**
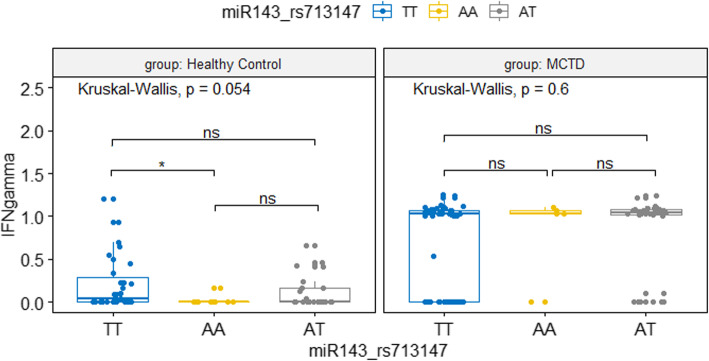
IFN-γ serum concentration between miR-143 rs713147 genotypes. HC group (N_AA_ = 10, N_AT_ = 25, N_TT_ = 31); MCTD group (N_AA_ = 5, N_AT_ = 34 ,N_TT_ = 55). ns: *p* >  0.05; *p ≤ 0.05; **p ≤ 0.01; ***p ≤ 0.001; ****p ≤ 0.0001; IFNgamma, IFN γ concentration in serum

**Fig. 2:**
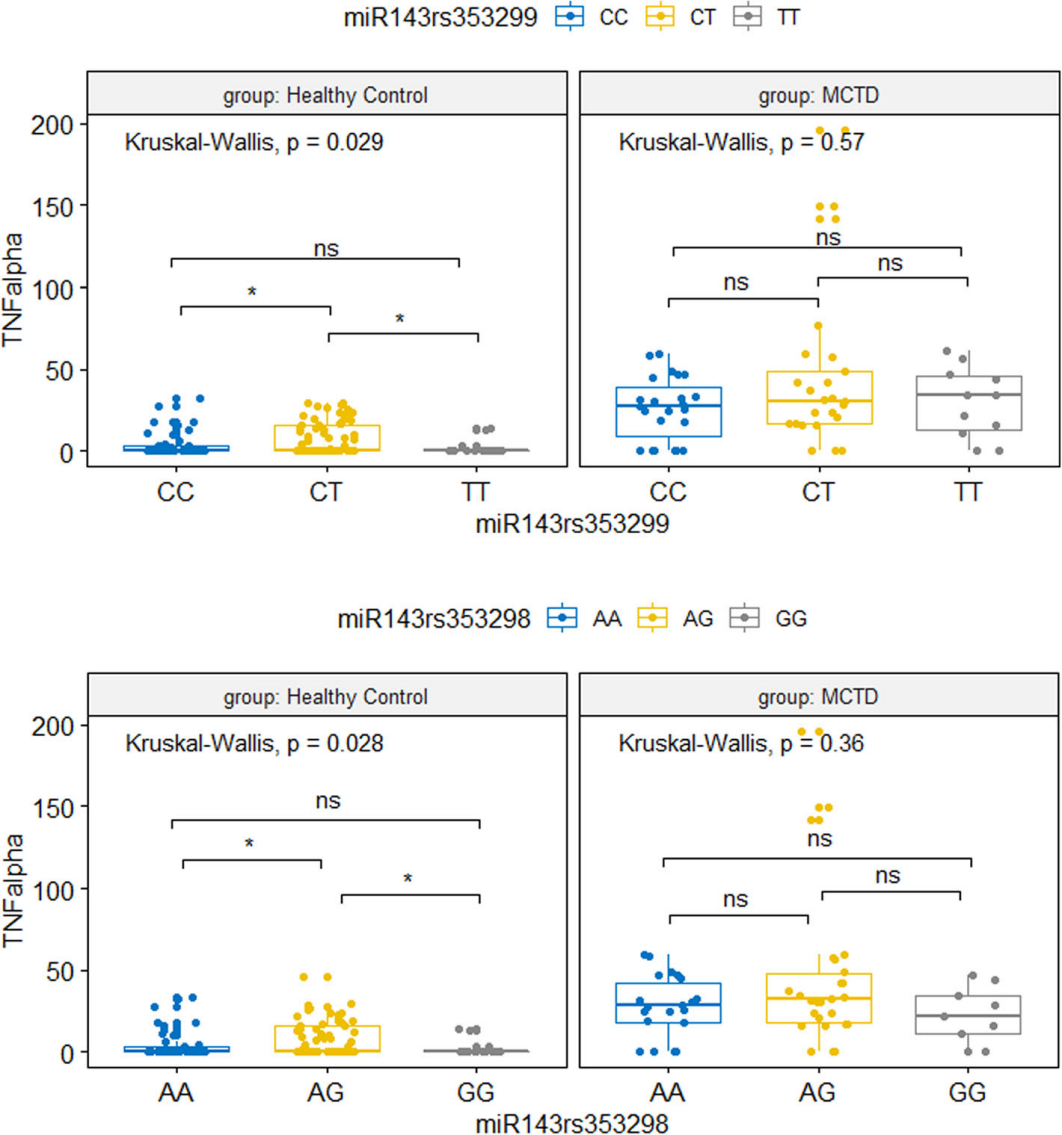
TNF-alpha serum concentration between miR-143 genetic variants. Upper plot: miR-143 rs353299 control group (N_CC_ = 47, N_CT_ = 66, N_TT_ = 15); MCTD group (N_CC_ = 23, N_CT_ = 25, N_TT_ = 11); bottom plot: miR-143 rs353298 control group (N_AA_ = 46, N_AG_ = 67, N_GG_ = 14); MCTD group (N_AA_ = 22, N_AG_ = 26, N_GG_ = 9); ns: *p* > 0.05; * *p* ≤ 0.05; **p ≤ 0.01; ***p ≤ 0.001; ****p ≤ 0.0001; TNFalpha, TNF-α concentration in serum

Second, we performed an analysis of the association between miR-155, miR-146a, and miR-143 genotypes and serum TNF-α, IFN-γ, and IL-6 levels in MCTD patients and control groups. Our results demonstrated a significant interaction between TNF-α, IFN-γ, and miR-143 genetic variants. Serum levels of IFN-γ were significantly higher within healthy subjects carrying miR-143 rs713147 TT genotype (*p* = 0.05, *ε*^2^ = 0.09, Fig. 1). However, in the group of MCTD patients, we did not observe any differences in serum levels of IFN-γ between miR-143 rs713147 genotypes (*p* = 0.6, *ε*^2^ = 0.01, Fig. 1). Analysis of TNF-α showed that miR-143 rs353299 CT and miR-143 rs353298 AG genotypes in healthy subjects were associated with higher TNF-α levels in serum (*p* = 0.03, *ε*^2^ = 0.06; *p* = 0.03, *ε*^2^ = 0.06; respectively, Fig. 2). In the MCTD patients, the TNF-α serum levels were generally higher, but we did not observe differences between individual miR-143 rs353299 and miR-143 rs353298 genotypes (*p* = 0.57, *ε*^2^ = 0.02; *p* = 0.36, *ε*^2^ = 0.04; respectively). In case of IL-6, we found no significant correlation between miRNA genotypes and IL-6 serum levels among both study groups, MCTD patients and healthy subjects.

### Association between miR-146a SNP distribution and TRAF6 and IRAK1 mRNA expression in whole blood

Distribution analysis indicated that only miR-146a may be associated with an increased risk of developing MCTD in our population. As we have known, miR-146a regulates immune and inflammatory signaling through TRAF-6/IRAK-1 pathway [30]. Based on these reports, we wondered whether miRNA-146a polymorphisms may affect the level of TRAF6 and IRAK1 gene expression. In the present study, the mRNA expression of the TRAF6 and IRAK1 were examined in the whole blood of 45 MCTD patients and 49 healthy subjects. We observed that mRNA expression of IRAK1 as well as TRAF6 were higher in the MCTD patients compared to controls, but only in the case of TRAF6, this difference was statistically significant (Supplementary Figure 2). In our study, we did not find an association between miR-146a SNPs and TRAF6/IRAK1 mRNA expression in both examined groups.

### miRNA expression in the sera of MCTD patients and healthy subjects

To evaluate the serum miR-143, miR-155, and miR-146a expression, we performed the analysis of the miRNA expression in 21 MCTD patients and 36 controls. We observed that serum miR-143, miR-155, and miR-146a expression were higher in MCTD patients compared to healthy subjects; however, these differences were not significant (Supplementary Figure 3).

## Discussion

The pathogenesis of autoimmune diseases is very complex, and many factors contribute to the abnormal functioning of the immune system.

Despite the growing knowledge of the importance of epigenetics in the regulation of immune response and the development of autoimmune diseases, there is still little research which relates to overlapping syndromes such as MCTD. Our research is the first study that examined the miRNA gene polymorphisms as well as expression in patients with MCTD. We investigated the association between the key in immune-regulation miRNAs: miR-155, miR-143, and miR-146a and MCTD.

Distribution analysis revealed that out of all microRNAs studied only miR-146a rs2910164 C allele may result in a genetic predisposition to MCTD. We observed a slight tendency where “CC + GC” (vs GG) were more characteristic for the MCTD group. These results are in line with Jazdzewski et al. [31] who showed that the rarer miR-146a rs2910164 C allele (found in the pre-miR-146a sequence) reduces the amount of pre- and mature 146a. Moreover, a meta-analysis in the Caucasian population showed an increased risk of developing autoimmune diseases in people carrying the miRNA-146a rs2910164 GC and CC genotypes [32]. It is suspected that the miRNA-146a rs2910164 C allele presence contributes to predispositions to AIDs by altering the expression of miR-146a; it is also possible that the miRNA-146a rs2910164 C allele changes the targets of miR-146a-3p. Research using EMSAs and luciferase reporter assays confirmed that the presence of this allele interferes with the binding of nuclear proteins like TRAF6 or IRAK1. A Shao et al. study on sepsis also confirms that the presence of the miRNA-146a rs2910164 CC genotype affects the expression of miR-146a and is associated with increased expression of IRAK1 and TRAF6 [33]. In the present study, TRAF6 and IRAK1 expression analysis does not confirm these observations. It may result from the fact that among our patients, only 4 were carriers of the miRNA-146a rs2910164 CC genotype. Accordingly, to our result, the miRNA-146a rs2910164 CC genotype was significantly more common among MCTD patients with scleroderma. These results emphasize the possible significance of the miRNA-146a rs2910164 CC genotype. Surprisingly, enlarged lymph nodes occurred more frequently in the group of MCTD patients with the miRNA-146a rs2910164 GG genotype (39% vs 12% of patients). This result is questionable, considering that the miRNA-146a rs2910164 GG genotype does not affect the expression of mature miR-146a which as a consequence should decrease the inflammatory response. Association studies have indicated that SNPs in miR-146a may affect the susceptibility of autoimmune diseases such as SLE, psoriatic arthritis (PsA), asthma, systemic sclerosis, AS, or telangiectasia. However, there are contradictions. Niu et al. also cite other studies (on the subject of AS, PsA, RA, or SLE) where the significance of the miR-146a SNPs in these diseases was not observed [34]. Although our observation showed a link between miR-146a genetic variant and MCTD onset, data do not allow to draw a clear conclusion and should be replicated. Functional studies are necessary to confirm the direct role of this genotype in the pathogenesis of MCTD.

miR-143 is well-known for its tumor-suppressive activity. Its expression prevents chronic inflammation and limits the invasion and migration of tumors [35]. Association analysis between miR-143 genotypes and MCTD incidence did not show any significant differences. However, the analysis of haplotypes showed that two of them, TTTG and CCTA, were more characteristic for the MCTD group (31% vs 15% and 23% vs 11% respectively). Interestingly, both haplotypes consist of alternative alleles of the tested miR-143 genetic variants (except the rs713147 T allele, which occurs in both haplotypes). Moreover, our results indicate a relation between single variants of the miR-143 gene and clinical symptoms like swollen fingers or hands, pericarditis/pleuritis, and scleroderma. We have demonstrated associations between examined miR-143 gene polymorphisms and higher serum concentrations of proinflammatory cytokines in the healthy group but not in the MCTD patients. We suspect that even if these miR-143 genetic variants are important, there must be other factors that contribute more significantly to the concentration of these cytokines in serum.

Although the participation of miR-155 in autoimmune disease development has been suggested in many works, our observations do not show that it has a significant role in MCTD susceptibility. There were no associations between clinical symptoms or inflammatory cytokine serum concentration. These observations exclude the miR155 SNPs significant importance in the development of MCTD in the Polish population. The microRNA expression in serum of MCTD patients did not differ significantly from the control group. However, the results were characterized by a large variation within groups, and therefore, we refrain from drawing conclusions based on them.

The limitations of this study include difficult biological materials and patients with different stories and treatment algorithms, which are difficult to diagnose. A small group of patients and the fact that we do not have detailed clinical information for some patients may affect the results and determine the low power of the study. The fact that MCTD is a rare complex disease makes the recruitment of a large sample size challenging. We also have to address the fact that our analysis was not normalized by all confounders. Moreover, testing of expression in serum instead of sorted specific cell lines may “dilute” cell-specific expression pattern. On the other hand, miRNAs located in tissues and body fluids like plasma or serum make them potential “blood-based biomarkers” of disease development or in predicting therapeutic responses. Nevertheless, expression research should be continued with a larger homogeneous group of patients in the same phase of the disease to be able to state unambiguous conclusions. Functional studies are necessary to provide a direct link between these miRNA and MCTD pathogeneses. Moreover, not only healthy controls but patients with different autoimmune disorders should be included in the study.

The MCTD disease entity is still a big challenge for clinicians in diagnosing and treating patients. Its rare occurrence and patient diversity additionally hinder research and thus a better understanding of the underlying cause of this disease. We believe that our study can be used as a base, or as part of a larger study or meta-analysis, which will show the differences or similarities between MCTD and other ACTDs, which in the future will allow us to unequivocally state the existence or exclusion of MCTD as a separate disease entity and facilitate its diagnosis.

## Conclusion

In conclusion, our study adds novel information in explaining the genetic and epigenetic predisposition among MCTD patients. We present preliminary association studies which indicate miR-146a and miR-143 as potential factors related to this disease. We believe that our findings may help in understanding the genetics and epigenetics of autoimmune processes not only in MCTD but also in other autoimmune diseases. However, we emphasize that these studies can provide only a direction for further research or be a comparison for similar, bigger studies of this rare disease entity.

## Supplementary Information

**Additional file 1.**

## Data Availability

The datasets used and/or analyzed during the current study are available from the corresponding author on reasonable request.
