# Supplementary material for: Association study between immune-related miRNAs and mixed connective tissue disease
Source: Arthritis Res Ther. 2021 Jan 11;23:19. doi: 10.1186/s13075-020-02403-9 (PMC7802256; doi:10.1186/s13075-020-02403-9)
Supplement: Supplementary file 1 — Additional file 1. [file 13075_2020_2403_MOESM1_ESM.docx]

**Supplementary Table 1.** SNPs information and genotyping results for MCTD patients and control groups.

| **SNP ID** | **Allele** | **SNP Type** | **MAF** | | | **p (HWE)** | |
| --- | --- | --- | --- | --- | --- | --- | --- |
|  |  |  | MCTD | Control | 1000 genome EUR, | MCTD | Control |
| miR-155 rs1893650 | C/T | Intron | 0.23 | 0.21 | 0.24 | 0.15 | 0.90 |
| miR-155 rs2829806 | G/T | Intron | 0.22 | 0.22 | 0.26 | 0.59 | 0.58 |
| miR-146a rs2910164 | G/C | Intragenic | 0.24 | 0.19 | 0.22 | 0.21 | 1 |
| miR-143 rs353291 | T/C | Intragenic | 0.39 | 0.40 | 0.38 | 0.33 | 1 |
| miR-143 rs353298 | A/G | Intron | 0.39 | 0.38 | 0.38 | 0.45 | 0.44 |
| miR-143 rs353299 | C/T | Intragenic | 0.39 | 0.37 | 0.38 | 0.71 | 0.48 |
| miR-143 rs713147 | T/A | Intron | 0.27 | 0.34 | 0.33 | 0.81 | 0.90 |

MAF - minor allele frequency; HWE - Hardy-Weinberg equilibrium; EUR - European;


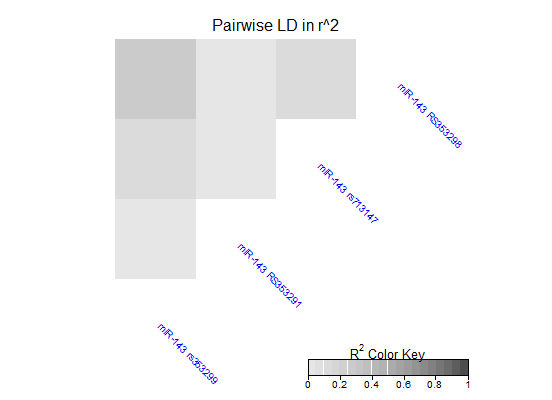


Supplementary Figure 1 Pairwise LD between miR143 genetic variants

**Supplementary Figure 2.** Relative quantification of TRAF6 and IRAK1 mRNA expression in the peripheral blood of MCTD patients (n=45) and healthy subjects (n=49). The expression of genes were determined by qRT‐PCR in the whole blood. Expression levels were normalized to control group (healthy subjects).


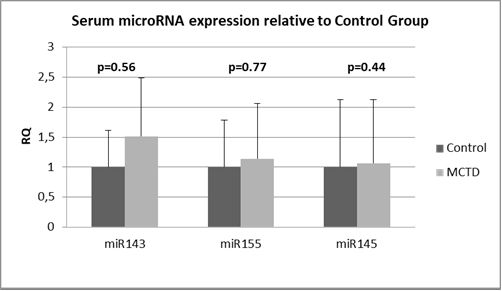


**Supplementary Figure 3.** Serum microRNA expression in MCTD compared to controls. miR-143, miR-155 and miR-145 expression were assessed in 21 patients with MCTD and 36 healthy subjects (control group). Serum microRNA expression is reported as delta delta cycle threshold (ΔΔCt). No significant differences between patients and Control group were determined (all p<0.05).
